# Metabolism, Ketosis Treatment and Milk Production after Using Glycerol in Dairy Cows: A Review

**DOI:** 10.3390/ani10081379

**Published:** 2020-08-08

**Authors:** Robert Kupczyński, Antoni Szumny, Katarzyna Wujcikowska, Natalia Pachura

**Affiliations:** 1 Department of Environment, Animal Hygiene and Welfare, Wrocław University of Environmental and Life Sciences, Chełmońskiego 38C, 50-375 Wrocław, Poland; k.wujcikowska@gmail.com; 2 Department of Chemistry, Wroclaw University of Environmental and Life Science, Norwida 25, 50-375 Wrocław, Poland; antoni.szumny@upwr.edu.pl (A.S.); natalia.pachura@upwr.edu.pl (N.P.)

**Keywords:** dairy cows, glycerol, metabolism, ketosis, rumen microorganism

## Abstract

**Simple Summary:**

Glycerol, as well as being an attractive feed ingredient for cattle, is also a by-product of a wide range of industrial applications. Glycerol has potential value in farming since it improves metabolism, feed efficiency, and can alleviate the symptoms of ketosis. Data indicate that glycerol can be a suitable partial grain replacement in the diet of cows during the transition period and at the beginning of lactation. The impact on milk yield is not significant, but glycerol mostly decreases milk fat content. The inclusion of dietary glycerol in the ration of dairy cows has an affect on ruminal fermentation patterns. Glycerol is rapidly fermented in the rumen into propionate, and it is metabolized to glucose in the liver through the process of glycogenolysis. Additionally, glycerol administration to ruminants can reduce greenhouse gas emissions. The purpose of this review is to highlight the potential benefits and drawbacks related to the use of glycerol in cattle.

**Abstract:**

The aim of this paper is to review and systematize the current state of knowledge on glycol metabolism in cattle. Glycerol, derived from biodiesel production, must be purified in order to be a useful product for feeding livestock. The use of glycerol in the feeding of ruminants can be justified for several reasons: (i) it is a source of energy in the ration, (ii) it is a glucogenic precursor, and (iii) it may have an effect on milk composition. The high energy value of glycerol provides the opportunity to use this raw material as a partial grain substitute in cattle feed rations. Dietary supplementation of glycerol is associated with increased propionate, butyrate, valerate, and isovalerate concentrations in the rumen. Glycerol can be used at up to 10%–15% of the dietary dry matter (DM) and is well-established as a treatment for ketosis in cows. Glycerol increases plasma glucose and may reduce non-esterified fatty acids and β-hydroxybutyrate levels. The use of glycerol does not have a clear effect on DM intake, milk yield, or milk composition. However, some authors have reported an increase in milk yield after glycerol supplementation associated with decreased milk fat concentration. It is also possible that the concentration in the milk of odd-chain fatty acids and *cis*-9, *trans*-11 conjugated linoleic acid may increase after glycerol application.

## 1. Introduction

The global production and use of environmentally friendly fuels is steadily increasing, a trend that has been particularly evident over the last two decades [1]. Worldwide interest in the production of renewable energy has focused attention on the production of biodiesel by the transesterification of vegetable oils. A significant by-product of the refining process is crude glycerol. In addition to market demand, this has also been driven by the creation of laws promoting green initiatives and the co-financing of research projects by EU agencies as well as governmental funding in developed countries [2]. The aim of these efforts is to use, exploit, and further develop modern technology regarding bio-based products from glycerol (G), as well as to develop new ways of using crude glycerol. Nevertheless, some social and environmental concerns exist in relation to biodiesel production [3]. The rapid growth of the biodiesel industry has resulted in an overproduction of crude glycerol. The most popular method of producing biodiesel is by transesterification of mainly vegetable or occasionally animal lipids, which produces the final by-product glycerol at a rate of approximately 200 g for every kg of biodiesel [4], while bioethanol production generates an additional 100% of glycerol [5]. Every 10 kg of fatty acid methyl esters (biofuel) produced generates approximately 1 kg of crude glycerol as a final by-product. This indicates that a 113,550 m^3^ per year plant can generate around 11,500 tonnes of 99.9% pure glycerol [6].

The purification of crude glycerol into a pure chemical substance (to be used in cosmetics, soaps, and food additives) is a relatively expensive process [5]. “Dirty” glycerol from biodiesel production must be purified in order to be a useful product for the food, pharmaceutical, and cosmetics industries. In previous years, crude glycerol can be given to ruminants as a source of energy, replacing grain in the diet without the need for thorough purification [7,8,9,10]. Currently, this also applies to glycerol as a feed additive. As the amount of glycerol available increases, so the price of glycerol decreases significantly, which encourages many farmers to use it in animal nutrition. When using glycerol in livestock feed, it is necessary to purify it, with only 1% of impurities allowed in the form of catalysts, salts, and methanol, which in larger quantities can be toxic to animals [9].

Glycerol is the main component of triglycerides, found in animal fat and vegetable oil. Glycerol is the simplest of the alcohols and is known as propane-1,2,3-triol. As a commercial product, it is also available as glycerol (1,2,3-propanotriol, trihydroxypropane, glyceritol, or glycidicalcohol). Physically, glycerol is a water soluble, viscous, odorless, colorless liquid and is characterized by a sweet taste [5].

In animal nutrition, high-purity glycerol should be used. Based on the European Commission regulation 68/2013 [11], crude glycerol is included in the list of feed materials. Crude glycerol may contain up to 0.5% methanol and up to 4% of matter organic non-glycerol, comprising fatty acid methyl esters, fatty acid ethyl esters, free fatty acids, and glycerides. Biodiesel production includes the transesterification of oils and fats of unspecified vegetables, with subsequent refining of the glycerol. Minimum glycerol content must be 99% of dry matter. Levels of methanol higher than those permitted could have serious health effects on calves, in which the rumen has not yet fully developed. When feeding glycerol to ruminants, a number of factors should be considered: (i) energy supply along with the feed ration, (ii) glycerol is a glucogenic precursor, and (iii) it may have an impact on milk production and composition. However, from the point of view of animal nutrition, a single dose of glycerol used in ruminants is limited by physiological factors and currently, to a lesser extent, its price. However, the sweet taste of glycerol is a factor that, in addition to its glucogenic properties, may have a beneficial effect on the increase in feed intake. In cattle production, glycerol is used as source of energy [7,10] and grain substitution in the diet, as well as in ketosis prevention and treatment. Therefore, this review summarizes findings from studies conducted using glycerol in cattle and its metabolism, especially its effect on rumen microbes, physiological parameters, feed intake, and milk production in dairy cows and ketosis treatment.

## 2. Effect of Glycerol on Rumen Processes and Metabolism

As mentioned in the Introduction, glycerol is a chemical compound that provides an increased source of energy for ruminants; moreover, it is an essential structural component in the biosynthesis of triglycerides and phospholipids. Glycerol has a significant effect on rumen fermentation patterns (Table 1), easily fermented in the rumen, and the propionic acid produced from it plays a key role in glucose production in the liver [12]. It is also included in the hepatic gluconeogenesis pathways by being absorbed from the rumen or the small intestine [13,14]. The first studies to determine the proportion of volatile fatty acids formed in the rumen were conducted by Wright [15]. He conducted an incubation with carbon 14-labeled glycerol, whose final metabolic products were acetic acid (31.4%), propionic acid (20.6%), carbon dioxide (17%), butyric acid (7.6%), lactic acid (3.5%), and others. Glycerol disappears rapidly from the rumen; however, it is not clearly indicated how much glycerol is absorbed from the rumen and how much is converted into propionate. More than 80% of administrated glycerol disappeared within 2 h of administration in steers [16]. This has been confirmed by more recent studies that indicate that the absorption of propionates into the bloodstream and the metabolic action is fast, and metabolic bottlenecks for propionate metabolism occur that might affect feeding behavior [17].

More than half a century ago, the efficacy of both propylene glycol (PG) and glycerol in the treatment of ketosis was confirmed [18]. Despite the usefulness of glycerol in improving hepatic gluconeogenesis, in the 1970s [19], glycerol was used primarily as a therapeutic agent. It was not until this century that its nutritional and therapeutic use was given a second life with the increase in production and the development of multicomponent formulations.

Dairy cows, when in negative energy balance (NEB), mobilize body fat reserves in the form of non-esterified fatty acids (NEFA) and glycerol. Cows with a milk yield of 30 kg take in around 100 g of glycerol from absorbed triglycerides from the diet [20]. The release of glycerol into the body occurs in two ways: during the hydrolysis of lipoproteins in the blood and during the lipolysis of fat reserves. Glycerol is only used in two organs: the liver and the mammary gland [20].

**Figure 1:**
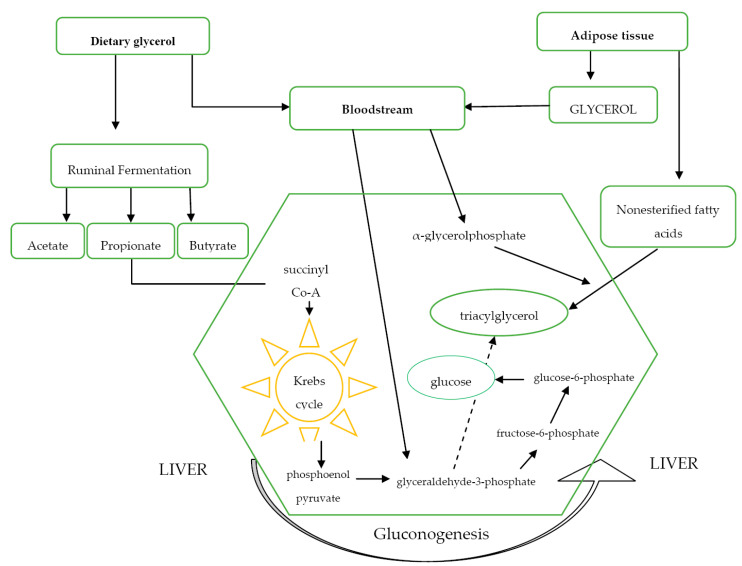
Pathways of glycerol transfer/metabolism and liver gluconeogenesis in dairy cows, modified from Osman et al. [12].

During the periparturient period in dairy cows, due to reduced dry matter intake, the demand for propionate and glucogenic amino acids from the rumen increases significantly [21]. In cases where gluconeogenesis is deprived of a suitable amount of substrates, cows will develop ketosis and hepatic steatosis syndrome. Osman et al. [12] suggested that glycerol, a glucogenic precursor, can be used for both gluconeogenesis and glycolysis. The effect of glycerol on the metabolism of cows is presented in Figure 1. Considering the above, it can be concluded that glycerol administered in feed or by drenching is converted to glyceraldehyde-3-phosphate [12]. Similarly, Goff and Horst [14] suggested that glycerol enters the gluconeogenic pathway at the level of dihydroxyacetone phosphate and glyceraldehyde-3-phosphate. When co-administered with glucagon [12] or in a larger amount [14], glycerol is likely converted to fructose-1,6-bisphosphate, which then supplies glucose through gluconeogenesis. Several studies have confirmed that feeding glycerol to cows increases the level of propionate in the rumen [7]. Glycerol also increased the level of acetyl coenzyme A (AcCoA) in the liver by 32% compared to propionates in one study [21]. Therefore, it is likely that propionic acid absorbed from the rumen decreases liver AcCoA content by stimulating its oxidation in the tricarboxylic acid cycle (TCA). The difference between glycerol and propylene glycol (PG) is that PG, being absorbed intact and entering the TCA cycle as pyruvate after its conversion to lactate, increases oxidation of AcCoA [22].

The administration of glycerol may be related to the increase in the expression of cytosolic phosphoenolpyruvate (PEPCK-C) mRNA during transition to lactation and suggests that the dietary energy source alters hepatic expression [23]. This increase in expression is important because PEPCK-C is a key enzyme for gluconeogenesis in the liver [24]. White et al. [23] show that the increase in PEPCK-C may indicate the regulation of hepatic gene expression by changes in rumen propionate production. Furthermore, in studies on sheep, it was found that glycerol supplementation upregulated stearoyl-CoA desaturate (SCD1) over five-fold in the liver [25].

The method of administration of glycerol as well as its quantity and purity influence the rumen environment. This influence, however, is also determined by the type of diet. For example, Kholif [26] suggests that decreased dietary content and intake of crude protein (CP) when feeding glycerol implies less protein availability for ruminal degradation. Microorganisms in the rumen adapt quickly to glycerol so that, after its administration, it quickly disappears from the rumen. Kristensen and Raun [27], when administering large amounts of glycerol (925 g/d), found that only 10% of this compound was found in the vena porta and the rest reached the liver as volatile fatty acids. Other studies show that glycerol can be absorbed from the rumen in significant amounts and its absorption mainly occurs by passive diffusion [28].

The use of glycerol (99.5%) as a replacement for maize resulted in significantly higher butyrate, valerate, and isovalerate concentrations in the rumen of Holstein cows [29]. At the same time, with increasing doses of glycerol, the concentration of acetate was significantly lower than in the control (Table 1). This has also been confirmed by other studies, which found that an increase in the concentration of butyrates in the volatile fatty acid pool is at the expense of a decrease in acetate concentration [30]. DeFrain et al. [8], administering glycol as a topdressing, both at a lower (0.43 kg/d) and a higher (0.86 kg/d) dose, reported an increase in total volatile fatty acids in rumen fluid, greater molar proportions of propionate, and a decreased ratio of acetate to propionate. It has also been shown in other studies that, with an increase in the dose of glycerol, a decrease in acetate and an increase in propionate was observed [31]. Similarly, when supplementing steers with glycerol (200 or 300 g of glycerol/d), an increase in propionate in the rumen was observed along with a decrease in the ratio of propionic to acetic acids [30]. This was also confirmed in studies by Rico et al. [32], who found that substituting corn starch with dry glycerol linearly increased propionate and valerate at the expense of acetate (Table 1). In these in vitro studies, an increase in neutral detergent fiber (NDF) digestibility was also found, but without a clear effect on the flow or efficiency of bacterial protein synthesis after increasing the level of glycerol. Rico et al. [32] indicate that glycerol as a dry product can replace dietary starch as corn starch at a level of up to 8% of DM in the diet without negatively affecting ruminal fermentation and digestibility during continuous culture (in vitro). In contrast to these studies, Donkin et al. [33] showed a tendency for glycerol therapy to reduce NDF digestibility, but the response was non-linear. NDF digestibility was reduced by 5% with an addition of 5% and 10% glycerol. Contrastingly, dry glycerol can effectively replace dry-rolled corn in diets for beef heifers when fed at 15% of diet DM, improving organic matter (OM) digestion without adversely affecting NDF digestibility [34].

Partially replacing concentrated ingredients in corn silage or cottonseed hull resulted in similar changes in the proportions of volatile fatty acids in the rumen [35]. Additionally, when introducing glycerol into the cow’s diet, efficiency of N utilisation was improved, as evidenced by lower concentrations of blood urea nitrogen and ruminal ammonia-N [35]. Administering glycerol in a mineral (zeolite) medium, a slight increase in pH and propionic acid content and a significant increase in butyrate after 3 h from administration of the preparation were observed only at a higher dose [36]. There was an increase in NDF degradation, as well as in organic matter and total protein.

In the ruminal fluid, starch and sugars are metabolized by *Selenomonas ruminantium* and Succinivibrio dextrinosolvens [37]. On the other hand, glycerol is metabolized by *Megasphaera elsdenii*, *Streptococcus bovis*, and *Selenomonas ruminantium* [38]. Glycerol supplementation in ruminants should significantly increase the amount of these bacteria in the rumen. Lactic acid is produced quite rapidly following the commencement of in vitro fermentation of glycerol and increases for up to 8 h of incubation [39]. A link exists between *M. elsdenii* and the increased concentration of butyric acid in the rumen fluid, which M. elsdenii produces from lactic acid [35]. *M. elsdenii* also produces propionate. In other studies, the abundance of *Megasphaera elsdenii* doubled after the use of crude glycerol (80% glycerol), but without a noticeable effect on rumen protozoa [40]. The presented changes in the rumen fluid microbiome explain the most frequently observed dynamics and directions of volatile fatty acid (VFA) changes.

Supplementation with crude glycerol to the diet generated a greater ruminal abundance of *Prevotella, Succinivibrio*, *Ruminococcus*, *Syntrophococcus*, and *Succiniclasticum* [41]. It also increases the abundance of butyrate-producing bacteria (e.g., *Pseudobutyrivibrio*) and *Selenomonas*, which is a bacterium capable of fermenting glycerol and is considered a secondary fermenter, converting lactate and glucose to propionate or valerate [41].

AbuGhazaleh et al. [29] showed that substituting 15% of the dietary corn with glycerol had no substantive effects on fermentation processing or ruminal bacteria. Only higher doses of glycerol caused a reduction in *Butyrivibrio fibrisolvens* and *Selenomonas ruminantium*. Glycerol may have interfered with *Butyrivibrio fibrisolvens*’ adhesion to feed particles, making nutrients less accessible to the bacterial cells. The DNA concentration for *Selenomonas ruminantium* was significantly lower at higher doses of glycerol, which may indicate that, unlike maize, glycerol supplementation results in a reduction in starch and sugar availability [29]. The inclusion of glycerol in the diet of young bulls did not affect the total bacterial count of Butyrivibrio fibrisolvens and Butyrivibrio proteoclasticus [42]. Madrid et al. [42] indicate that the high levels of glycerol (80 g of glycerol per kg of DM) cause a decrease in the ruminal pH (Table 1), despite the increase in *Selenomonas ruminantium.*

## 3. The Effect of Glycol on Physiological Parameters in Relation to Ketosis

During the transition period and at the beginning of lactation, there is a sharp increase in demand for the energy and nutrients required for fetal development, as well as colostrum and milk production, which is accompanied by hormonal changes [45,46]. The decrease in dry matter (DM) intake in multiparous cows may amount to 31% in the period from the 21st to the 1st day of antepartum [47]. In addition, feed intake is significantly lower in cows exhibiting overconditioning during the dry period [47,48]. Energy deficiency and/or decreased feed intake during the periparturient period result in increased lipolysis of deposited fat and the release of NEFA into the blood [47,48]. An excessive increase in NEFA concentration leads to the accumulation of triglycerides (TG) in the liver and a significant increase in ketonic compound production [49]. The pool of AcCoA in the liver is continually replenished by β-oxidation of NEFA and the entry of other fuels through the pyruvate, but AcCoA is particularly abundant for cows in the postpartum period that are in a lipolytic state [21]. The addition of glucogenic precursors to the food ration may reduce the energy deficit or shorten its duration, while acting antilipolytically and antiketogenically. In practice, this is very important because the frequency of subclinical ketosis at the beginning of lactation can be as high as 40% in dairy cows [50]. 

In many studies [30,51,52,53], it has been found that a gluconeogenic precursor might be effective in the treatment or prevention of ketosis. Table 2 summarizes the effects of glycerol on physiological parameters in blood. The administration of glycerol results in an increase in plasma glucose, either by administering it in the feed or as a drench. Plasma glucose responses increased with greater glycerol dosages for a longer time [14]. In this study [14], the following were used during treatment: 1, 2, and 3 L of glycerol in 9.5 L of water via an esophageal pump, and after just 0.5 hr, an increase in blood glucose concentration of 16%, 20%, and 25%, respectively, was observed. Nielsen and Ingvartsen [54] demonstrated that the LD50 of a similar glucogenic compound, propylene glycol (PG), is 2.2 kg PG for a cow weighing 600 kg. In the study by Goff and Horst [14], when administering 3 L of glycerol, staggering and depression were observed in two out of three cows. These symptoms disappeared within 4 h. Importantly, there were no significant changes in the pH of the rumen. On the other hand, the sulfur-containing gases produced during propylene glycol fermentation in the rumen may contribute to the toxic effects seen in cattle when high doses are administered for therapeutic purposes [39]. In young bulls that were fed high levels of concentrate, glycerol at 20 or 40 g/kg of DM could be included without affecting the ruminal pH or raising the propionic acid, but at 80 g/kg, the ruminal pH dropped to 5.74 vs. 6.32 in a glycerol-free diet [42].

Oral administration of pure glycerol for 14 days after calving resulted in a significant increase in glucose concentration on day 7 postpartum and triacylglycerols on day 1 postpartum [12]. At the same time, during the first 14 days after calving, plasma glucagon and NEFA decreased, while there was a decrease in plasma β-hydroxybutyrate (BHBA) on day 1 postpartum. Osman et al. [12] recorded that the increase in blood glucose occurs within the first 4 h after oral glycerol administration and remains elevated for a further 8 h. The rate of change in blood glucose levels in cows has been confirmed by Goff and Horst [14]. In the study carried out in young crossbred dairy bulls, it was found that an increase in the dose of glycerol results in a linear increase in blood glucose and in average daily weight gain [55]. Administering glycerol (270 mL/d) in sheep showed an increase in glucose and insulin after 30 min, and high glucose levels persisted for up to 360 min after oral drenching and up to 720 min for insulin [56]. However, the insulin response to glycerol lasted longer than that of propylene glycol and molasses.

In our own studies, we compared the effect of short-term (7 day antepartum to 7 day postpartum) administration in cows of propylene glycol and glycerol (99% pure) in two forms: as an addition to the total mixed ration (TMR) (topdressed) and oral drenching [53]. Both additives were administered at 300 mL/d. Propylene glycol and glycerol restricted the reduction in BHBA at 5 days postpartum. In the control group, this buildup was significant. On the final day of supplementation, the highest concentration of glucose was found in the groups that received glycerol. On the other hand, this glucogenic effect of glycerol is weaker than that typically expected when propylene glycol is drenched [57].

Kass et al. [58] evaluated an oral drench of 500 g of glycerol (82.6% pure) administered once daily before feeding during the first 3 weeks postpartum and observed a decrease in plasma NEFA concentration early in lactation. In addition, when pure glycerol was given topdressed to cows in early lactation (from 4 to 63 d), a linear increase in glucose and decreased NEFA and BHBA were found [30]. Concentrations of glucose in plasma were higher for cows fed glycerol relative to control (54.1 vs. 58.1 mg/dL, respectively) and linearly increased with increasing glycerol supplementation levels (100, 200, and 300 g per cow). A significant increase in glucose levels was also found after the application of unprotected fish oil with glycerol [59]. These studies also indicate a beneficial effect of the applied supplementation on the activity of enzymes, especially gamma-glutamyl transferase (GGT). In Simmental cows, which are less susceptible to metabolic disorders during the perinatal period, the supplementation of glucogenic precursors (glycerol or propylene glycol) also caused an increase in glucose concentration, but hepatic enzyme secretion did not increase after supplementation with both agents [60]. In addition, the infusion of glycol (98.7%) into the abomasum resulted in increased plasma glucose and insulin concentrations [21]. On the basis of experiments by Piantoni and Allen [22], 300 mL of propylene glycol administered to the rumen is more effective than glycerol or a combination of both.

In other studies, glycerol was administered to drinking water at 20 g/L for 7 days antepartum until 7 days postpartum [52]. In these studies, no glucogenic effect was found in such a short period of time, but after calving, the concentration of BHBA decreased. DeFrain et al. [8] recorded a decrease in blood glucose levels and an increase in BHBA when adding glycerol to TMR. Higher doses of glycerol resulted in a slight decrease in NEFA in the blood and increased insulin levels. In the case of short-term (4 d) administration to cows of boluses of compounds constituting gluconeoglucogenic precursors, it was found that a 300-mL dose of propylene glycol is more effective at increasing plasma glucose concentration than glycerol and at least as effective as 600 mL of glycerol or a combination of the two when administered in the cranial reticulorumen [22].

Use of glycerol in powder form (65% of food grade glycerol) in the transition period resulted in an improvement in energy status underlined by a higher concentration of plasma glucose, lower concentrations of plasma BHBA, and lower concentrations of urine ketones [61]. The glucogenic effect did not cause a statistical effect on milk yield or feed intake in the first 3 weeks of lactation; however, the yield of cows receiving glycerol was 52 kg/d, whereas in control cows, it was 46 kg/d. A moderate antilipolytic and glucoplastic effect of glycerol in powder form was found by Farkašová et al. [36], using a glycerol-containing preparation at a dose of 300 g/d. In studies by Bodarski et al. [62], no clear effect on metabolic changes at the beginning of lactation was observed in cows administered glycerol in powder form (430 or 860 g/d), but an effect on milk production was observed. According to Piantoni and Allen [22], propylene glycol decreased dry matter intake (DMI) compared with glycerol, which might indicate that propylene glycol increased the oxidation of AcCoA. On the contrary, glycerol did not, which is consistent with the hepatic oxidation theory [63].

## 4. Effect of Glycerol Administration on Milk Production and Composition

There are few works that have determined the energy value of glycerol for ruminants. DeFrain et al. [8] reported an energy value of 1.91 Mcal NEL/kg for glycerol. It contains about 4.32 Mcal/kg gross energy and 2.27 Mcal/kg net energy for lactation (NEL) [65]. In other studies, the calorific value of glycerol ranged from 1.98 to 2.26 Mcal NEL/kg [10]. Impurities present in glycerol affect its calorific value. The energy value of pure glycerol can roughly be considered to be equivalent to that of maize grain. The energy estimates for glycerol are higher with low starch diets. However, the energy value of glycerol depends on the energy density of the ration, the level of glycerol nutrition, and interactions with other components of the ration [9]. The energy value is reduced by 13% when glycerol is added to starch-rich rations due to the reduced digestibility of neutral detergent fiber [10]. Glycerol (86% glycerol) could be assigned an metabolisable energy (ME) estimate of 3.47 Mcal/kg of DM when fed to Holstein bulls receiving high-concentrate diets [43].

A number of studies have been carried out on the effect of glycerol on production parameters when administered to dairy cows [33,53,66], beef cattle [43,55,67], goats [68], and lambs [69]. In sheep studies, it has been established that a positive effect on growth rates, feeding behavior, and blood metabolites is achieved with no higher than 4.7% DM [69]. Replacing 7.5% of alfalfa hay in a beef cattle diet with crude glycerol can be beneficial to animal performance, boosting final BW and ADG, where it increased from 0% to 7.5% glycerol [67]. However, 10% glycerol supplementation had already had a negative impact on production parameters. The apparent digestibility of nutrients increased with increasing glycerol in the diet, for similar feed intake and different levels of glycerol in the ration [70]. The composition of the basic ration has a great influence on the possibility of introducing glycerol into the diet of feedlot calves. Ramos and Kerley [71] demonstrate that crude glycerol addition to a diet did not negatively affect ruminal fermentation, and the addition of up to 20% in concentrate and hay-based diets should not affect performance or carcass characteristics.

Table 3 summarizes the effects of PG on milk production and milk composition in dairy cows. Administering a glucogenic mixture including 70% glycerol, 20% propylene glycol, and 10% water in sheep showed that, apart from a beneficial effect on glucose metabolism, milk yield and lactose content decreased, while protein and casein content increased significantly [72]. In studies on dairy cows (Table 3), similar results have been obtained, although without any decrease in milk yield [64]. A number of studies indicate that it is possible to administer crude glycerol up to 15% DM to dairy cows without negative effects on milk yield [33,64,66]. The commonly used doses of glycerol do not significantly affect DMI, milk yield, or milk composition in dairy cows [33,53]. Donkin et al. [33] showed that cows fed the 15% glycerol diet had decreased DMI for the first 7 days of the experiment. Supplementation of excessive amounts of glycerol (30%) to the food ration admittedly improved feed efficiency, but it reduced DMI, which linearly decreased to 3.5% fat-corrected milk (FCM), with a tendency to reduce milk yield [64].

Increasing the density of energy in the diet of lactating cows can help to increase milk yield. Omazic et al. [66], administering glycerol from the second day after calving to the fourth week of lactation, observed that cows fed high-purity glycerol (99.5%) tended to have higher milk yield than cows receiving low-purity (88.1%) (35.5 vs. 33 kg/d). Similar trends were observed when drenched orally with crude glycerol [58]. However, a number of studies indicate that glycerol has no significant effect on the milk yield of cows [33,52,53,64]. Partially replacing ground corn, corn gluten feed, and citrus pulp with crude glycerol at 5% of dietary DM increased DMI without increasing milk yield [35]. Concentration and production of milk fat and apparent total-tract digestion of dietary NDF were reduced when crude glycerol was fed at a higher dose (10% of DM). However, DeFrain et al. [8] reported tendencies for a lower milk fat yield and milk urea nitrogen when glycerol was given. Osborne et al. [52] supplemented glycerol in drinking water and found that it had no effect on milk yield or composition. The administration of glycerol to Simmental cows also did not demonstrate a clear effect on milk yield or composition [60]. On the other hand, Bodarski et al. [62], administering glycerol in powder form as a topdressing (300–500 g/d) to cows from 3 weeks antepartum to 70 days postpartum, observed a significant increase in productivity and protein content in the first 10 weeks of lactation. Glycerol improves nitrogen utilisation efficiency, which can increase milk protein content and protein yield [33].

An additional goal of using glycerol is the possibility of enriching milk with odd-chain fatty acids and cis-9,trans-11 C18:2 conjugated linoleic acid (CLA). Milk fat odd- and branched-chain fatty acids originate principally from both ruminal amylolytic bacteria and de novo synthesis in the mammary gland [73]. Ezequiel et al. [64] found a linear increase in the concentration in milk of C15:0, C17:0, cis-9,trans-11 C18:2 (CLA), C20:4n-6, and C22:4n-6 when supplementing with up to 30% glycerol. For the highest dose of glycerol, there was a comparable increase in CLA, due to the use of polyunsaturated fatty acids (PUFAs) contained in fish oil [74]. On the other hand, when administering glycerol, no significant increase was observed in Butyrivibrio fibrisolvens, a bacteria that hydrogenates and isomerates cis-9,cis-12 C18:2 [75]. Combined administration of 300 mL/d of unprotected fish oil and 150 mL/d of glycerol did not have a significant effect on milk yield but had a noticeable effect on increasing the levels of cis-9, trans-11 CLA, and n-3 FA in milk [59]. The increase in eicosapentaenoic (EPA) and docosahexaenoic (DHA) acids concentration found in this study was similar to the concentration of these fatty acids after the administration of protected fish oil.

The use of glycerol supplementation may reduce the fat content of milk [8,31,58]. With increasing doses of glycerol, this reduction in fat content can be linear [76]. A reduction in milk fat concentration is generally related to the diet composition, e.g., a low forage to concentrate ratio, high starch diets, and high PUFAs. Specific dietary compositions may modify the biohydrogenation pathways of fatty acids in the rumen by producing a number of intermediate isomers [77]. C18:2 trans-10,cis-12, a CLA isomer formed during the isomerisation of C18:2n-6 in the rumen, is an inhibitor of milk fat synthesis in mammary glands of dairy cows [77]. Additional studies are required in order to clarify the relevance of all CLA isomers formed in the rumen, as well as trans C18:1 isomers influenced by glycol, and the relationship between the formation of these FAs and the fat content of milk.

Glycerol administered to TMR during the periparturient period did not affect the body condition score (BCS) of the cows [8]. Additionally, the administration of glycerol to drinking water had no effect on changes in BCS [52]. The administration of glycol as a topdressing has a greater inhibitory effect on the decline in the condition of cows immediately after calving than drenching [53].

## 5. Conclusions

Numerous studies have examined the effect of glycerol administration and dose on physiological changes occuring in cattle. Dietary glycerol is beneficial to the dairy cow because it appears to increase ruminal propionate, thereby increasing the supply of gluconeogenic substrate to the liver. However, as the dose of glycerol increases, the abundance of *Butyrivibrio Fibrisolvens* and *Selenomonas ruminantium* in the rumen decreases. Used as a partial replacement for grain, glycerol has a high calorific value, similar to that of maize. Economics will dictate whether this replacement is feasible. Glycerol could be used at up to 10% (max. 15%) of the dietary DM without negative effects on cow performance. This approach can reduce the costs of overall production, both by lowering the costs of using grain and by the prophylactic effect of glycerol on the risk of metabolic diseases. Its glucogenic properties are manifested when it is metabolized in the rumen into propionate and when it is absorbed and metabolized in the liver. For this reason, glycerol is also highly effective in the treatment of ketosis. Additional studies are required to clarify the relevance of CLA and *trans*-C18:1 isomers formed in the rumen upon glycerol supplementation and the relationship between the formation of these FAs and milk fat depression syndrome.

## Figures and Tables

**Table 1:** The effect of glycerol on the fermentation, microbiome, and pH in the rumen of cattle.

Purity	Method of Administration and Dose		Cows and Days in Milk	Time of Sampling	Rumen pH	Acetate (Molar%)	Propionate (Molar%)	Butyrate (Molar%)	Acetate/ Propionate	Reference
Glycerol, 80.2%	Topdressed and hand-mixed into the upper 1/3 of the daily ration	0	Holstein cows From 14 d prepartum to 21 d in milk	4 h after feeding	6.91	61.8	21.7	12.3	2.85	DeFrain et al. [8]
		0.43 kg/d			6.89	55.4	27.1	14.4	2.04	
		0.86 kg/d			6.61	58.5	24.7	13.2	3.27	
Glycerol, 86%	Mixed with TMR	0	Holstein bulls (335 ± 8.6 kg of initial BW). Suplementation until 91 d of the study	Monthly during 2 consecutive days	6.07	54.6	35.6	9.8	1.53	Mach et al. [43]
		4% of DM			6.06	54.1	34.3	11.6	1.57	
		8% of DM			5.68	50.9	38.6	10.5	1.32	
		12% of DM			6.08	53.3	35.4	11.3	1.50	
Glycerol, 99.5%	Mixed with grain	0	Holstein fistulated cows	3 h after the morning feeding	6.53	38.51	19.14	12.83	2.01	AbuGhazaleh et al. [29]
		15%			6.54	36.81	18.59	16.98	1.98	
		30%			6.57	33.65	19.65	19.41	1.71	
		45%			6.53	33.54	22.91	18.23	1.46	
Glycerol (crude glycerol 80.1%)	Glycerol mixed with TMR	0% DM (based diet corn silage)	Holstein cows 116 ± 13 DIM. 27 d periods for each diet	8 h after feeding	6.15	57.0	20.6	12.0	2.8	Shin et al. [35]
		10% DM (based diet corn silage)			5.99	48.0	26.9	13.9	1.82	
		0% DM (based diet cottonseed hulls)			6.25	52.5	25.2	12.6	2.13	
		10% DM (based diet cottonseed hulls)			6.18	46.8	28.4	14.0	1.72	
Glycerol (dry glycerol)	Replacing corn starch with glycerol in TMR	0	Lactating cow	During continuous culture	6.5	56.8	22.6	14.5	2.5	Rico et al. [32]
		3% (0.75 kg/d)			6.5	50.9	23.3	18.4	2.2	
		5% (1.25 kg/d)			6.4	49.4	27.6	15.8	1.8	
		8% (2 kg/d)			6.5	44.5	30.3a	17.1	1.5	
Glycerol, 80%–85%	Mixed and the coated cottonseed added to the TMR	0	Holstein cows 56 ± 18 DIM, fistulated cows	0, 2, 4, 6, 8, and 10 h after feeding	6.1	61.1 ^a^	59.1 ^b^	57.6 ^c^	2.7	Boyd et al. [31]
		200g/d			6.0	23.3 ^a^	24.1 ^b^	25.4 ^c^	2.5 ^b^	
		400g/d			6.1	11.4 ^a^	12.3 ^b^	12.4 ^b^	2.3 ^c^	
Glycerol (crude glycerol 86%)	Glycerol added to food	0% DM	Ruminally cannulated Nellore steers averaging 24 months of age and 400 kg BW	Collected on d 15 of each experimental period, at 1, 0, 2, 4, 6, and 8 h after feeding	6.39	86.6	24.5	19.3	3.65	Van Cleef et al. [44]
		7.5% DM			6.11	73.4	22.3	21.0	6.56	
		15% DM			6.20	59.9	24.1	28.8	2.82	
		22.5% DM			6.27	54.1	29.9	24.6	1.92	
		30% DM			6.23	49.3	33.1	30.5	1.53	
Glycerol	Offered in a mash twice daily	0	Bulls (Limousin x indigenous Spanish breed) of 292.8 ± 29.6 kg of BW	0, 2, 4, and 8 h (date of mean) after the morning feeding	6.32	60.5	24.1	10.6	3.03	Madrid et al. [42]
		20 g/kg of DM			6.38	61.0	26.7	9.14	2.55	
		40 g/kg of DM			6.14	58.3	29.0	8.69	2.13	
		80 g/kg of DM			5.74	52.8	29.7	13.1	1.96	

TMR—total mixed ration; DM—dry matter. Mean values in the same row with different superscripts differ significantly (*p* < 0.05).

**Table 2:** The effect of gycerol administration on concentrations of blood glucose, insulin, non-esterified fatty acids (NEFA), and β-hydroxybutyrate (BHBA) in cows.

Purity/Method of Administration	Dose	Experimental Design (Time of Use)	Time of Sampling or Day	Glucose (mmol/L)	Insulin (µIU/mL)	NEFA (μEq/L)	BHBA (mmol/L)	Reference
Glycerol, 80.2%/topdressing	Control	Holstein cows 14 d prepartum to 21 d postpartum	21 d postpartum, 4 h after feeding	3.70	11.65	624	0.24	DeFrain et al. [8]
	0.43 glycerol kg/d			3.50	12.20	639	0.34	
	0.86 glycerol kg/d			3.32	12.28	495	0.29	
Dry glycerol/topdressing in TMR	Control	From first 3 weeks of lactation	2 h after the morning feeding on 4, 7, 14, and 21 d of parturition	3.07	5.18	349	0.89	Chung et al. [61]
	250 g/d (162.5 g of glycerol/d)			3.08	4.75	371	0.83	
Crude glycerol (82.6%)/oral drench	Control	Primiparous Holstein dairy cows 4 to 21 of lactation	Before the administration of glycerol (data 3 period aplication)	4.73	6.28	634	0.63	Kass et al. [58]
	glycerol 500 mL/d			4.63	5.49	702	0.88	
Crude glycerol (82.6%)/topdressed with corn silage	Control	Cannulated multiparous Holstein cows (114 ± 29 DIM), 3 weak	2 h after morning feeding) on d 21	3.53	-	-	-	Ezequiel et al. [64]
	15% of DM			4.24				
	30% og DM			3.75				
Glycerol (≥99.5 %)/One bolus infusion per d in cranial reticulorumen.	300 mL of PGl	Cannulated multiparous Holstein cows (22 ± 5 DIM)	4 days. Blood samples before infusion and at 10, 20, 30, 40, 50, 60, 80, 100, 120, 150, 180, 240 min, and 24 h postinfusion. (Present date maximum value—baseline value).	2.86	1.73	484	17.6	Piantoni and Allen [22]
	300 mL of G			2.86	1.66	501	16.8	
	600 mL of G			2.88	1.52	599	14.9	
	300 mL of G + 300 mL of PG			2.93	1.39	536	13.5	

G—glycol; PG—propylene glycol.

**Table 3:** The effect of glycerol on milk yield and milk composition in dairy cows.

Purity and Dose		Method of Administration	Cows and Time of Suplementation	Milk (kg/d)	Fat (%)	Protein (%)	ECM ^a^ (kg/d)	FE	Reference
Glycerol, 80.2%	0	Topdressed and hand-mixed into the upper 1/3 of the daily ration	Holstein cows, 14 d prepartum to 21 d pospartum.	37.2	4.26	2.94	38.7	-	DeFrain et al. [8]
	0.43 kg/d			35.7	4.02	3.02	35.2		
	0.86 kg/d			34.0	4.26	2.97	35.0		
Dry glycerol	0	Topdressed in TMR	From parturition to 3 weeks into lactation	42.14	4.37	3.19	41.72	1.91	Chung et al. [61]
	250 g/d			44.57	4.00	3.09	42.04	2.07	
Glycerol, 80.1%	0% DM (corn silage-based diet)	Glycerol mixed with TMR	Holstein Cows 116 ± 13 DIM. 27-d periods for each diet	34.4	3.10	2.88	29.6	1.19	Shin et al. [35]
	5% DM (corn silage-based diet)			35.6	3.20	2.86	31.3	1.25	
	10% DM (corn silage-based diet)			35.6	3.01	2.86	30.1	1.28	
	0% DM (cottonseed hulls-based diet)			37.7	3.15	2.95	32.8	1.19	
	5% DM (cottonseed hulls-based diet)			36.6	3.32	3.01	32.7	1.04	
	10% DM (cottonseed hulls-based diet)			36.4	3.05	3.00	31.3	1.07	
Glycerol, 80%–85%	0	Mixed and the coated cottonseed was then added to the TMR	Holstein cows 4 weeks treatment	37.9	3.46	2.76	37.6	1.55	Boyd et al. [31]
	200g/d			37.3	3.31	2.75	36.1	1.60	
	400g/d			35.5	3.35	2.81	34.6	1.51	
Crude glycerol, 82.6%	0 400 mL	Oral drench	4 to 21 d of lactation (date 3 period)		(kg/d)		-	-	Kass et al. [58]
				29.4	1.42	0.927			
				31.5	1.53	0.992			
Crude glycerol, 82.6%	0	Topdressed whit corn silage	21 days (7 d of data collections in each period)	17.9	3.17	3.03		0.95	Ezequiel et al. [64]
	15% of DM			16.9	2.71	3.05		0.95	
	30% og DM			16.0	3.01	3.24		1.11	
Glycerol, 99.5%	300 mL of PG	Infusion in rumen for 4 d	Cannulated multiparous Holstein cows (22 ± 5 DIM)	43.1	4.90	2.86			Piantoni and Allen [22]
	300 mL of G			42.3	4.48	2.97			
	600 mL of G			42.6	4.66	2.90			
	300 mL of G + 300 mL of PG			42.3	4.52	2.84			

ECM—energy corrected milk yield; FE—feed efficiency = milk yield (kg/d)/DMI (kg/d). Mean values in the same row with different superscripts differ significantly (*p* < 0.05).
